# Clinical symptom improvement and lipidomic signatures in overweight/obese PCOS treated by lifestyle and acupuncture intervention

**DOI:** 10.3389/fmed.2025.1642095

**Published:** 2025-10-24

**Authors:** Haolin Zhang, Wenmin Tian, Dong Li, Yang Chen, Yang Ye, Zejun Huo, Li Shi, Ii Fukuzawa, Shuhan Yang, Yang Yang, Hua Zhang, Lin Zeng, Xiyan Xin, Chunyong Wang, Wen Ma, Weiyu Qiu, Zhihui Qi, Heng Dai, Na Li, Haining Wang, Rong Li

**Affiliations:** ^1^State Key Laboratory of Female Fertility Promotion, Department of Traditional Chinese Medicine, Peking University Third Hospital, Beijing, China; ^2^Department of Obstetrics and Gynecology, National Clinical Research Center for Obstetrics and Gynecology, Center for Reproductive Medicine, Peking University Third Hospital, Beijing, China; ^3^Center for Precision Medicine Multi-Omics Research, Institute of Advanced Clinical Medicine, Peking University, Beijing, China; ^4^Department of Integration of Chinese and Western Medicine, Peking University Health Science Center, Beijing, China; ^5^Research Centre of Clinical Epidemiology, Peking University Third Hospital, Beijing, China; ^6^State Key Laboratory of Natural and Biomimetic Drugs, School of Pharmaceutical Sciences, Peking University, Beijing, China; ^7^Department of Endocrinology and Metabolism, Peking University Third Hospital, Beijing, China; ^8^Research Units of Comprehensive Diagnosis and Treatment of Oocyte Maturation Arrest, Chinese Academy of Medical Sciences, Beijing, China

**Keywords:** lifestyle and acupuncture intervention, polycystic ovary syndrome (PCOS), body mass index (BMI), insulin resistance, lipidomic

## Abstract

**Background:**

Polycystic ovary syndrome (PCOS) is interrelated with obesity. Lifestyle intervention, mainly focusing on weight loss, has emerged as promising strategies to improve fertility outcomes in women with obesity. Acupuncture can aid in weight loss through metabolic regulation. However, evaluating the effectiveness of lifestyle and acupuncture in improving symptoms of polycystic ovary syndrome is still lacking.

**Results:**

This study aims to evaluate the efficacy of long-term lifestyle and acupuncture treatment on PCOS and elucidate the lipidomic features of these interventions in patients with overweight/obese PCOS, and identify potential therapeutic targets. Thirty-one women with PCOS and twenty-eight age and BMI matched controls were recruited. The participants with PCOS were randomly assigned to either lifestyle and acupuncture intervention group (A&L) or Lifestyle and sham acupuncture group (SA&L) for 4 months. The patients were followed up for an additional 4 months. Clinical indices indicating hyperandrogenism, homeostasis and quality of life was collected to evaluate the effectiveness of treatments. Comprehensive lipidomic analyses by utilizing mass spectrometry were conducted to profile the lipidomic signatures of participants with PCOS before and after treatment. After the A&L intervention, the BMI of participants with PCOS was significantly reduced. Assessments of insulin resistance (HbA1c, HOMA-IR, ISI), hyperandrogenism (SHBG, FAI), physical activity, and *β*-endorphin showed significant improvement. Lipidomic analysis revealed a panel of lipid species that effectively distinguished participants with PCOS from those without PCOS (AUC = 0.9747). Serum 18:0 Lyso PI, involved in intracellular insulin signaling pathways, was specifically associated with the A&L regimen. The co-regulation of 18:0 Lyso PI and 16:0 SM (d18:1/16:0) were associated with the A&L regimen.

**Conclusion:**

18:0 Lyso PI may be a potential biomarker for A&L therapy in PCOS patients.

## 1 Background

Polycystic ovary syndrome (PCOS) is a prevalent condition affecting 8–13% of infertile women. PCOS has serious metabolic and reproductive health implications and is often associated with obesity (1, 2). Excess abdominal adipose tissue (AT) initiates metabolic and endocrine aberrations that are crucial in the progression of PCOS (3). Abdominal AT impairs insulin action, which contributes to the progression of hyperandrogenism. Excessive androgen levels, in turn, lead to impaired glucose uptake, further contributing to insulin resistance and increased visceral fat deposition. Pharmacological treatment of PCOS is not well-received due to side effect and lack of long-term evaluation (4). Lifestyle interventions, mainly focusing on weight loss, have emerged as promising strategies to improve fertility outcomes in women with obesity. Thus, lifestyle intervention and weight management are recommended as the first line treatment for PCOS (5). Acupuncture, a low risk non-pharmacological therapy, has gained recognition as an alternative treatment for weight loss with fewer adverse effects (6). It has been shown to regulate appetite, enhance energy expenditure, and contribute to weight loss (7). Additionally, acupuncture has demonstrated positive effects on anxiety symptoms and overall quality of life in individuals with obesity, potentially by influencing food intake and energy homeostasis (8). Our prospective pilot studies have shown that Lifestyle and acupuncture (A&L) treatment decreases body weight, increases whole-body glucose uptake in PCOS (9), and induces multiple metabolic changes related to BMI and insulin resistance (IR) in PCOS (10–12).

Lipidomic, the study of lipid profile changes in biological samples, offers valuable insights into the metabolic alterations associated with PCOS (13). Lipidomic studies have been conducted to explore differences in lipid profiles between participants with PCOS and controls, aiming to identify potential biomarkers and therapeutic targets for PCOS. Several lipid classes, including phosphatidylcholines (PCs), sphingomyelins (SMs) (14, 15), diacylglycerols, triacylglycerols, and free fatty acids (16), have been found to be altered in patients with PCOS. Specific combinations of PC subclasses and circulating free fatty acid levels have exhibited high diagnostic accuracy for PCOS (17). Moreover, lipidomic has been employed to investigate the impact of pharmacological interventions on lipid metabolism in PCOS, such as the use of metformin, a commonly prescribed medication for PCOS patients with insulin resistance, which has been shown to reduce ceramides, sphingolipids, and glycerophospholipid levels (18). However, no study to date has examined the relationship between specific lipid species and the efficacy of acupuncture intervention in women with PCOS who are overweight/obese.

Therefore, the objective of our study was to systematically explore the effects of lifestyle and acupuncture on metabolic parameters, and quality of life. Furthermore, we aimed to investigate the features of the lipid profile of participants with PCOS undergoing lifestyle and acupuncture treatment using an ultra-high-performance liquid chromatography-quadrupole time-of-flight mass spectrometry (UPLC-QTOF-MS)-based lipidomic approach, providing novel insights into the inter-relationships of complex metabolic between obesity and PCOS and how to manage the disease.

## 2 Methods

### 2.1 Study design and participants

The cohort consisted of 31 overweight or obese participants with PCOS, recruited from March 2016 to December 2021 at the clinical department and health center of Peking University Third Hospital. This cohort is part of the clinical trial (NCT04193371). Inclusion criteria were: female participants diagnosed with PCOS based on the Rotterdam criteria 2003 (19), with at least two of the following symptoms: infrequent ovulation or anovulation; hyperandrogenism or clinical manifestations of high blood androgen; ultrasound findings of polycystic ovaries in one or both ovaries, or ≥12 follicles measuring 2 to 9 mm in diameter, and/or ovarian volume ≥10 mL. Participants were aged between 20 and 40 years with a BMI ranging from 25 to 40. They had no immediate fertility wishes and were willing to use barrier contraceptive methods for 32 weeks and sign the consent form.

Exclusion criteria included having any other endocrine disorder (e.g., androgen-secreting tumors, thyroid dysfunction, diabetes); receiving pharmacological or acupuncture treatment within the past 3 months; or being pregnant or breastfeeding in the last 6 months.

Additionally, 28 age- and BMI-matched control participants without PCOS were also included for this study. Fasting serum samples were collected from participants in the follicular phase. Ethical approval was obtained from the Regional Ethical Review Board of Peking University Third Hospital (Beijing, China; approval number, PKU3-IRB-M2019021). All participants provided written informed consent.

### 2.2 Lifestyle and acupuncture treatment

16 eligible participants with PCOS were randomly assigned to the active acupuncture and lifestyle management (A&L) group, while the remaining 15 participants were assigned to the sham acupuncture and lifestyle management (SA&L) group. All participants received lifestyle advice starting from the baseline visit. The lifestyle intervention was assisted by a patent PCOS lifestyle management system (National invention patent, ZL 2015 10500978.9) (9), which combined the website, app and wearable device, via the Doctor-Regulation–Based web and mobile health intervention targeting an active lifestyle with a wearable device (20). A face-to-face nutritional consultation was subsequently arranged for each patient to tailor an individualized lifestyle treatment. Meanwhile, individualized nutrition and activity advice is provided to each patient in terms of their weight-loss goals and food diaries each week following WHO recommendations. All participants received a step-counter and upload a lifestyle App for daily use, a recipe for exercise and diet each day, and a physical exercise diary for daily reporting of exercise and diet: number of steps, type of activity, intensity, and time (minutes). Daily nutrient intake feed back to the researcher immediately.

**Figure 1:**
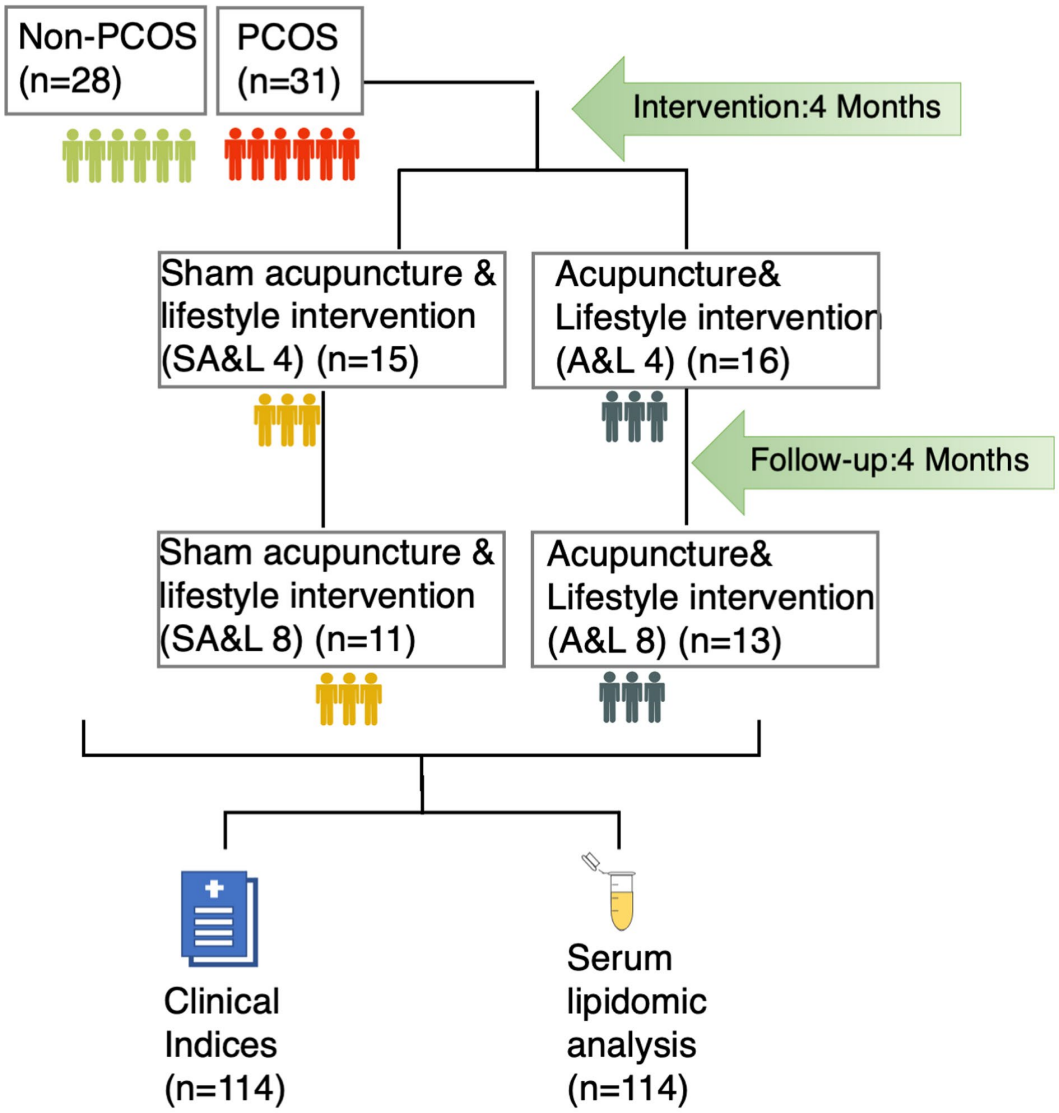
The study recruited 31 participants with PCOS and 28 age and BMI matched controls. Participants with PCOS were randomized into two treatment groups: acupuncture plus lifestyle intervention (A&L) for or sham acupuncture plus lifestyle intervention (SA&L), all for 4 months followed by 4 months follow-up. Clinical indices were evaluated and serum samples were collected at each time point.

The acupuncture intervention was administered three times per week for 30 min over a consecutive 4-month period, with a total of 32 to 48 sessions as previously described (21). Participants were followed up for another 4 months after the completion of the treatment period (Figure 1).

Two sets of 14 acupuncture points were selected, alternating between sets for each treatment session. Additionally, the acupuncturist could choose 2–3 additional acupoints based on Traditional Chinese Medicine (TCM) diagnoses and experience. To elicit the needling sensation (de qi), all points were manually stimulated by rotating the needle immediately after insertion. Electroacupuncture stimulation was applied using the HANS-200 device (Nanjing Jisheng Medical Technology Co., Ltd., China) with a frequency of 2 Hz and intensity ranging from 0.1 to 2.0 mA.

In the sham acupuncture group, needles were inserted superficially to a depth of <5 mm at non-acupuncture and non-meridian points in the shoulder or upper arm (22). Electrodes were attached to the needles, and the stimulator was turned to zero intensity.

### 2.3 Clinical indices measurements

#### 2.3.1 Glucose tolerance and insulin sensitivity

Glucose tolerance and insulin sensitivity were assessed using the oral glucose tolerance test (OGTT). The homeostatic model assessment of insulin resistance (HOMA-IR) was calculated as: [fasting insulin (μU/mL) × fasting glucose (mmol/L)]/22.5, while the HOMA-*β* (islet β-cell function) was calculated as 20 × fasting insulin (μU/mL)/[fasting plasma glucose (mmol/L) − 3.5] (11). The composite insulin sensitivity index (ISI) was calculated using the formula: 10000/square root of (fasting glucose × fasting insulin × mean glucose × mean insulin). Hyperinsulinemia was estimated by calculating the area under the curve (AUC) using the trapezoid method (23). Additionally, fasting blood samples were obtained to measure glycated hemoglobin (HbA1c).

#### 2.3.2 Hormonal profile

Sex hormones, including estrogen (E2), progestin (P), luteinizing hormone (LH), follicle-stimulating hormone (FSH), prolactin (PRL), testosterone (T), and androstenedione (A2), were measured using the Immulite 2000 immunoassay system (Siemens Healthcare Diagnostics, Germany). Sex hormone-binding globulin (SHBG) was measured using the Beckman DxI800 immunoassay system (Beckman Coulter, Brea, CA, USA). The free androgen index (FAI) was calculated using the formula: TES (nmol/L) × 100/SHBG (nmol/L). Serum anti-Müllerian hormone (AMH) concentrations were measured by an ultrasensitive two-site enzyme-linked immunosorbent assay (ELISA) (Ansh Laboratories LLC, Webster, TX, USA) (11).

#### 2.3.3 Lipidomic analysis

Serum samples were mixed with an extraction solvent (chloroform, 2:1), briefly vortexed, and sonicated for 1 h. They were then centrifuged using an Allegra 64R Centrifuge (BECKMAN COULTER, USA). The supernatant was used for mass spectrometry analysis. Lipid analysis was performed using high-performance liquid chromatography electrospray ionization on an AB SCIEX Triple TOF 5600 mass spectrometer. Liquid chromatography was conducted on an XBridge Peptide BEH C18 column (Waters, USA; 3.5 μm, 2.1 × 100 mm). Lipid extracts (2 μL) were injected and separated under a stepped gradient condition with a flow rate of 400 μL/min. Solvent A consisted of water: formic acid (99.9, 0.1), and solvent B consisted of isopropanol: acetonitrile: formic acid (49.95, 49.95:0.1). Both solvents contained 10 mM ammonium acetate. The gradient elution program started at 65% A, reached 20% A in 2 min, switched to and remained at 100% B from 9 to 15 min, and returned to 65% A from 16 to 20 min. Chromatographic data were qualitatively analyzed using PeakView1.2 according to the lipid standard database and quantified using MultiQuant2.1 (24).

### 2.4 Statistical methods

Pearson correlation analyses were conducted to examine the associations between BMI and other clinical indicators. t-tests were performed to determine differences between two groups, while comparisons among more than two groups were performed using analysis of variance (ANOVA) followed by Tukey’s post-hoc tests. Statistical analyses were conducted using Prism 8.0.2 (GraphPad, San Diego, CA, USA), with *p* < 0.05 considered statistically significant.

For the lipidomic analysis, missing values in the dataset were imputed with the mean value. Partial least squares-discriminant analysis (PLS-DA) was performed using MetaboAnalyst software (Wishart Research Group, University of Alberta, Edmonton, AB, Canada). t-test was conducted to generate a serum lipidomic panel to differentiate participants with PCOS from control participants, with a significance threshold of *p* < 0.05 after adjusting for BMI and an absolute log_2_ fold change greater than 0.30. The ROC panel was evaluated using a logistic regression model with leave-one-out (LOO) cross-validation.

The MEGENA R package was used to build correlation networks from differentially correlated lipid pairs in the two indicated groups. Differential correlation was calculated using the R package DGCA, and only lipid pairs with differential correlation (empirical *p*-value < 0.05) were included for further analyses.

## 3 Results

### 3.1 Lifestyle and acupuncture intervention reduced BMI and improved symptoms of PCOS

This study enrolled 31 participants with PCOS and 28 age- and BMI-matched control participants without PCOS. The participants with PCOS were randomized into two treatment groups: one receiving acupuncture plus lifestyle intervention (A&L) and the other receiving sham acupuncture plus lifestyle intervention (SA&L) for 4 months, followed by a 4-month follow-up period. Clinical indices and serum samples were collected to evaluate treatment effectiveness (Figure 1).

**Figure 2:**
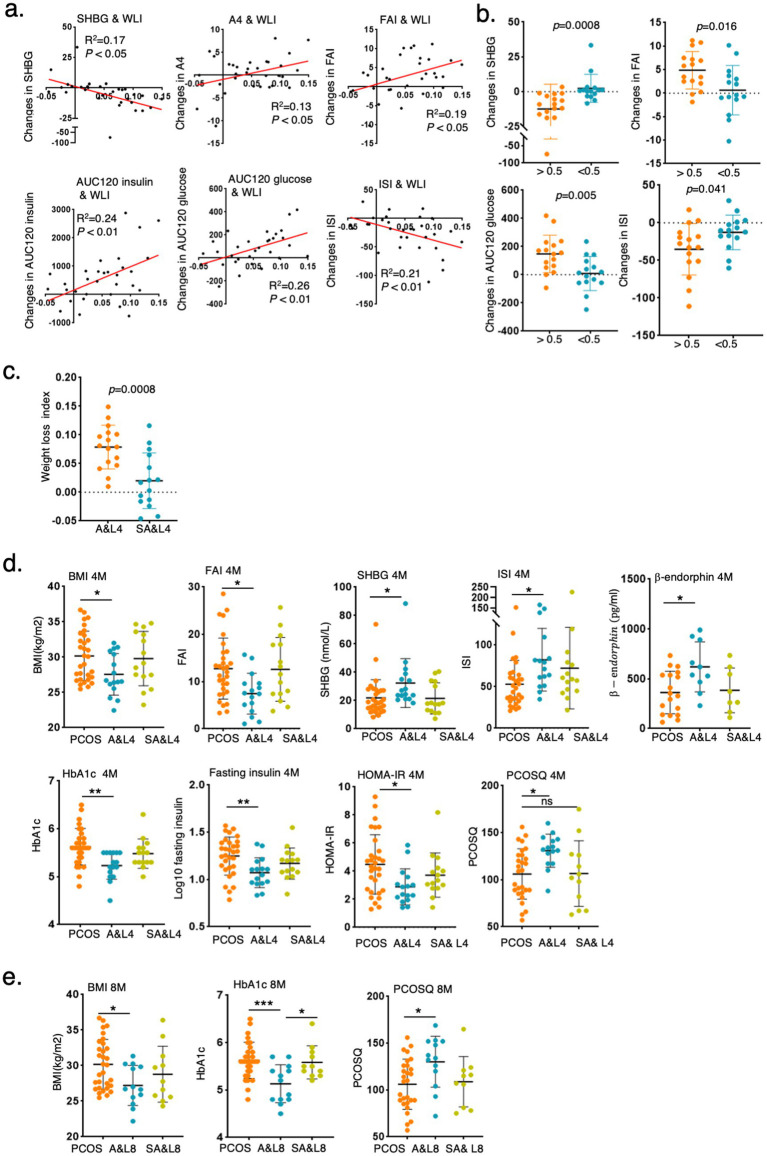
Lifestyle and acupuncture intervention reduced BMI and improved symptoms of PCOS. **(a)** Correlation analysis of WLI with delta changes in clinical indices after the 4-month intervention. (Weight loss index (WLI) = (BMI pre-intervention - BMI post-intervention)/BMI pre-intervention). **(b)** Changes in clinical indices in participants with PCOS grouped by WLI>0.05 or WLI<0.05 after 4 months of intervention. **(c)** Comparison of the WLI between the A&L intervention group and the SA&L intervention group. **(d,e)** Changes in clinical indices in participants with PCOS after A&L and SA&L interventions. The levels of clinical indices in participants with PCOS before intervention, 4 months after intervention **(d)**, and 8 months after intervention **(e)** are shown.

The clinical indices of the control participants and participants with PCOS before and after intervention are shown in Table 1. The BMI of participants with PCOS significantly decreased after the A&L intervention (27.57 ± 2.95 vs. 30.18 ± 3.48, *p* < 0.01), while no significant difference was observed in the SA&L group (Table 1). To eliminate the effect of initial BMI difference on weight loss, we defined a weight loss index (WLI) as the ratio of the difference between pre-intervention BMI and post-intervention BMI to pre-intervention BMI. Correlation analysis revealed positive associations between the WLI and improvements in androstenedione (A2), FAI, AUC120 glucose, and AUC120 insulin. Negative correlations were observed between the WLI and improvements in SHBG and ISI (Figure 2a).

**Table 1:** Characteristics and clinical indices of 59 participants.

Characteristics	Non-PCOS	PCOS	A&L	A&L	SA&L	SA&L	Non-PCOS VS PCOS	A&L 4 months VS PCOS	A&L 8 months VS PCOS	SA&L 4 months VS PCOS	SA&L 8 months VS PCOS
			4 months	8 months	4 months	8 months					
Age (years)	31.07 ± 5.36	28.45 ± 5.06	28.69 ± 5.24	29.23 ± 5.21	28.20 ± 5.03	29.45 ± 4.76	NS	NS	NS	NS	NS
BMI (kg/m^2^)	28.65 ± 2.53	30.18 ± 3.48	27.57 ± 2.95	27.01 ± 2.86	29.80 ± 3.84	28.79 ± 3.94	NS	*	**	NS	NS
Waist-hip ratio (%)	87.68 ± 5.49	87.30 ± 5.67	85.55 ± 5.98	85.11 ± 5.84	87.14 ± 6.08	87.00 ± 6.56	NS	NS	NS	NS	NS
Body fat rate (%)	40.11 ± 3.69	42.17 ± 5.23	38.81 ± 4.94	36.30 ± 6.75	40.02 ± 6.28	39.24 ± 6.76	NS	*	**	NS	NS
Lean weight	44.21 ± 3.66	44.30 ± 2.95	43.16 ± 2.51	43.87 ± 3.99	44.09 ± 3.28	44.51 ± 3.24	NS	NS	NS	NS	NS
HbA1c	5.50 ± 0.55	5.63 ± 0.38	5.23 ± 0.28	5.13 ± 0.40	5.48 ± 0.31	5.58 ± 0.35	NS	***	***	NS	NS
Fasting Glucose (mmol/L)	5.38 ± 0.76	5.11 ± 0.70	5.09 ± 0.40	5.30 ± 0.92	5.29 ± 0.56	5.35 ± 0.77	NS	NS	NS	NS	NS
Fasting insulin (mU/L)	19.06 ± 15.22	19.45 ± 8.14	12.59 ± 4.94	14.30 ± 6.95	15.81 ± 6.7	13.98 ± 8.72	NS	**	*	NS	*
HOMA-IR	4.95 ± 5.98	4.48 ± 2.11	2.89 ± 1.27	3.44 ± 1.84	3.72 ± 1.58	3.28 ± 2.08	NS	**	NS	NS	NS
HOMA-B	202.35 ± 108.34	394.76 ± 721.38	159.97 ± 50.87	174.44 ± 79.84	190.19 ± 92.59	172.92 ± 103.24	NS	NS	NS	NS	NS
AUC 120 glucose	988.93 ± 227.97	992.27 ± 144.40	886.78 ± 180.29	889.50 ± 113.50	938.80 ± 195.01	885.68 ± 106.37	NS	*	*	NS	*
AUC 120 insulin	11631.54 ± 7110.74	14242.35 ± 6696.57	10071.38 ± 4518.92	9842.00 ± 5320.59	10303.00 ± 5548.73	11265.00 ± 6883.02	NS	*	*	NS	NS
PRL ng/ml	11.73 ± 5.93	12.32 ± 8.39	12.70 ± 8.14	11.62 ± 6.08	14.37 ± 10.19	13.51 ± 11.74	NS	NS	NS	NS	NS
LH mIU/ml	4.50 ± 2.99	7.40 ± 4.66	5.82 ± 3.24	5.46 ± 3.10	5.78 ± 2.67	8.69 ± 4.42	**	NS	NS	NS	NS
FSH mIU/ml	6.79 ± 2.81	5.51 ± 1.49	6.31 ± 1.27	5.92 ± 1.01	5.44 ± 1.24	6.07 ± 1.99	*	NS	NS	NS	NS
E2 pmol/L	178.20 ± 50.77	200.19 ± 106.80	152.69 ± 38.86	142.00 ± 55.71	166.01 ± 56.38	161.54 ± 43.82	NS	NS	NS	NS	NS
P nmol/L	1.74 ± 2.95	1.04 ± 0.53	0.98 ± 0.40	0.92 ± 0.34	0.99 ± 0.36	0.90 ± 0.30	NS	NS	NS	NS	NS
A_2_ nmol/L	7.27 ± 3.28	10.13 ± 4.58	8.96 ± 4.84	8.36 ± 4.52	10.43 ± 4.50	10.46 ± 3.27	**	NS	NS	NS	NS
T nmol/L	1.55 ± 0.50	2.29 ± 0.82	1.99 ± 0.91	2.17 ± 0.95	2.19 ± 0.74	2.13 ± 0.71	***	NS	NS	NS	NS
FAI	6.29 ± 4.03	12.76 ± 6.46	7.46 ± 4.32	8.78 ± 5.21	12.60 ± 6.75	9.97 ± 4.23	****	**	NS	NS	NS
SHBG nmol/L	31.70 ± 14.64	21.86 ± 12.91	32.36 ± 17.22	29.16 ± 10.35	21.51 ± 10.97	24.13 ± 7.70	**	*	NS	NS	NS
AMH ng/ml	3.83 ± 3.59	7.62 ± 4.40	4.92 ± 3.88	6.12 ± 4.48	7.62 ± 5.78	10.37 ± 5.65	***	*	NS	NS	NS

*p* values comparing different groups have been computed using student’s t-test.

A, acupuncture; SA, sham acupuncture; L, lifestyle management; HbA1c, glycated hemoglobin; HOMA-IR, homeostatic model assessment of insulin resistance; HOMA-B, homeostatic model assessment of islet β-cell function; AUC, area under the curve; PRL, prolactin; LH, luteinizing hormone; FSH, follicle-stimulating hormone; E2, estrogen; P, progestin; A2, androstenedione; T, testosterone; FAI, free androgen index; SHBG, sex hormone-binding globulin; AMH, serum anti-müllerian hormone.

*p < 0.05, **p < 0.01., ***p < 0.001.

Participants with PCOS were further divided into two subgroups with the cut off value of 0.05 of WLI after 4 months of intervention. Significant differences were found in SHBG, FAI, AUC120 glucose, and ISI between these two groups. Participants with PCOS who experienced greater weight loss demonstrated better improvements in these indices (Figure 2b). Notably, 12 of the 16 participants in the A&L group had a WLI greater than 0.05 (Figure 2c).

After 4 months of intervention, the A&L group showed significant reductions in BMI, FAI, SHBG, glycosylated hemoglobin, fasting insulin, HOMA-IR, PCOSQ scores and *β*-endorphin, which is reflecting quality of life, significantly increased. These changes were not observed in the SA&L group (Figure 2d). At the 4-month follow-up, the A&L group maintained significantly lower BMI, glycosylated hemoglobin, and PCOSQ scores, comparable to levels at the end of the intervention (Figure 2e). Other clinical indices, such as FAI, SHBG, fasting insulin, and HOMA-IR et al., which showed significant improvements after the 4-month A&L intervention, remained improved compared to baseline at the 4-month follow-up (Supplementary Figure S1).

### 3.2 Lipidome characteristics of participants with PCOS

**Figure 3:**
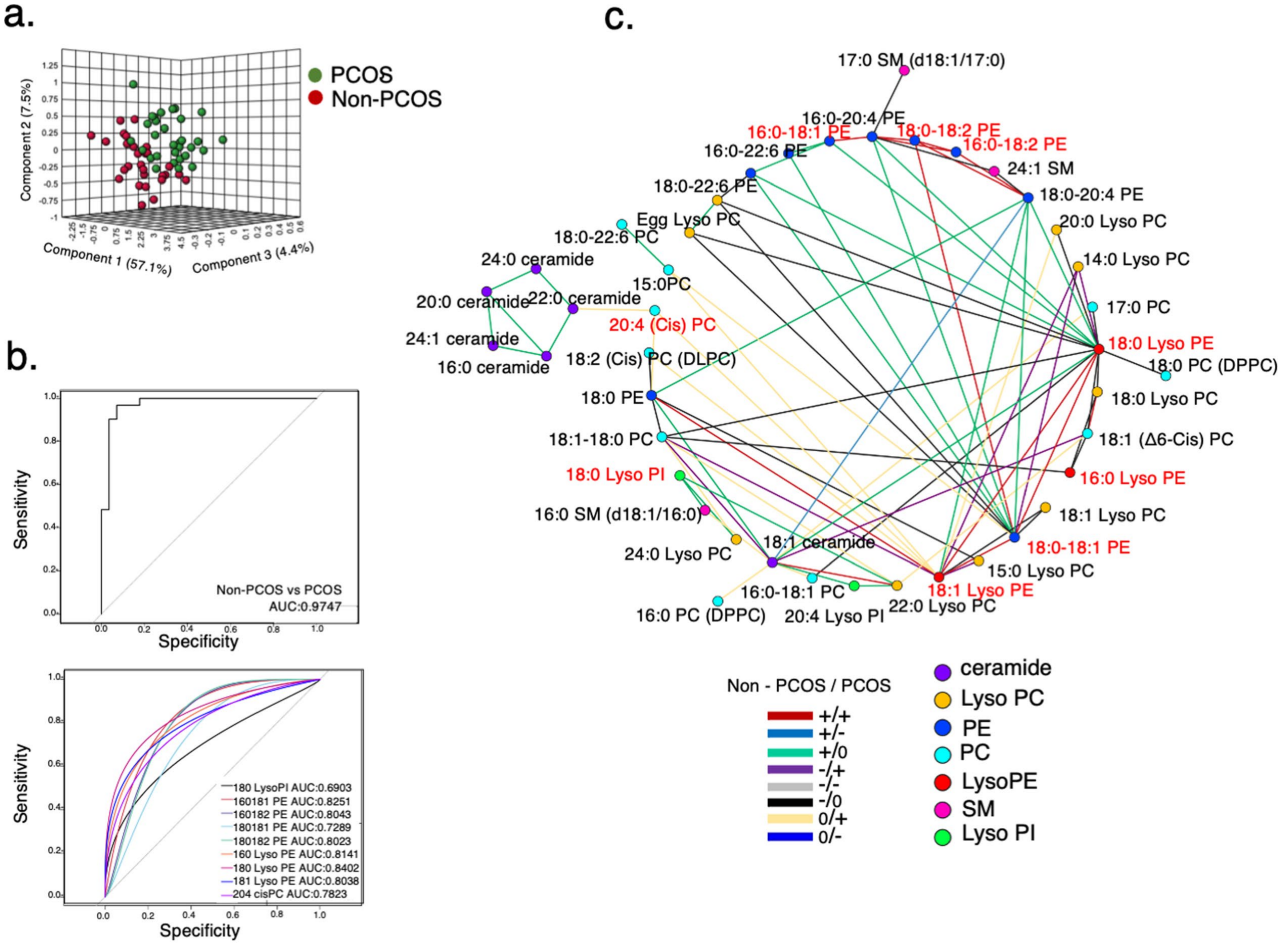
Lipidome characteristics of participants with PCOS. **(a)** PLS-DA model for distinguishing between the PCOS group and control group at baseline. A total of 44 metabolic variables were identified and used to establish the PLS-DA model. **(b)** Receiver operating characteristic (ROC) curves of differentially expressed lipids. A panel of nine lipids, selected using adjusted *p* < 0.05 and a fold change > 2 between participants with PCOS and controls. 18:0 Lyso PI, 16:0-18:1 PE, 16:0-18:2 PE, 18:0-18:1 PE, 18:0-18:2 PE, 16:0 Lyso PE, 18:0 Lyso PE, 18:1 Lyso PE, and 20:4 cisPC distinguished controls from participants with PCOS with an area under the curve (AUC) = 0.9747 in the logistic regression model. **(c)** Differential correlation analyses of plasma lipids in participants with PCOS compared to controls at baseline. Multiscale embedded correlation network analysis illustrates changes in lipid metabolic pathways. Only lipid pairs with significant differential correlations (empirical *p* < 0.05) are included. The positive or negative correlation is indicated by + or -. No significant correlation is indicated by 0. For instance, the green line (+/0) in the left lower legend indicates that the correlation between two connected lipid pairs is positive (+) in the controls. The correlation was not significant (0) in the participants with PCOS.

To explore the potential mechanisms of the A&L intervention on PCOS, the serum lipidome of 114 samples was analyzed. PLS-DA results showed that the identified lipids effectively differentiated between the non-PCOS control group and participants with PCOS before intervention (Figure 3a). Among these lipids, 29 differentially expressed lipids were identified between controls and PCOS after adjusting for BMI (*p* < 0.05). Nine of these differentially expressed lipids exhibited a fold change greater than 2, including mostly Lyso PEs, PEs, and 18:0 Lyso PI (Supplementary Figure S2). These nine lipids could distinguish controls from PCOS in a logistic regression model, with an AUC of 0.9747. Each of the nine lipids displayed a diagnostic value above 0.69 (Figure 3b).

Furthermore, the analysis of differentially correlated lipid pairs between non-PCOS controls and participants with PCOS revealed that 18:0 Lyso PI acted as a hub, connected to 16:0 SM (d18:1/16:0) and 22:0 Lyso PC through green lines (+/0) (Figure 3c). This indicates that the positive correlations between 18:0 Lyso PI and 16:0 SM (d18:1/16:0)/22:0 Lyso PC in controls were absent in participants with PCOS. Compared to non-PCOS controls, the relative intensity of 18:0 Lyso PI was significantly higher in participants with PCOS, whereas the relative intensities of 16:0 SM (d18:1/16:0) and 22:0 Lyso PC were significantly lower (Supplementary Table S1). This suggests that changes in the relative intensities of these lipids in participants with PCOS disrupted the correlation pattern.

### 3.3 18:0 Lyso PI is associated with lifestyle and acupuncture intervention in PCOS

**Figure 4:**
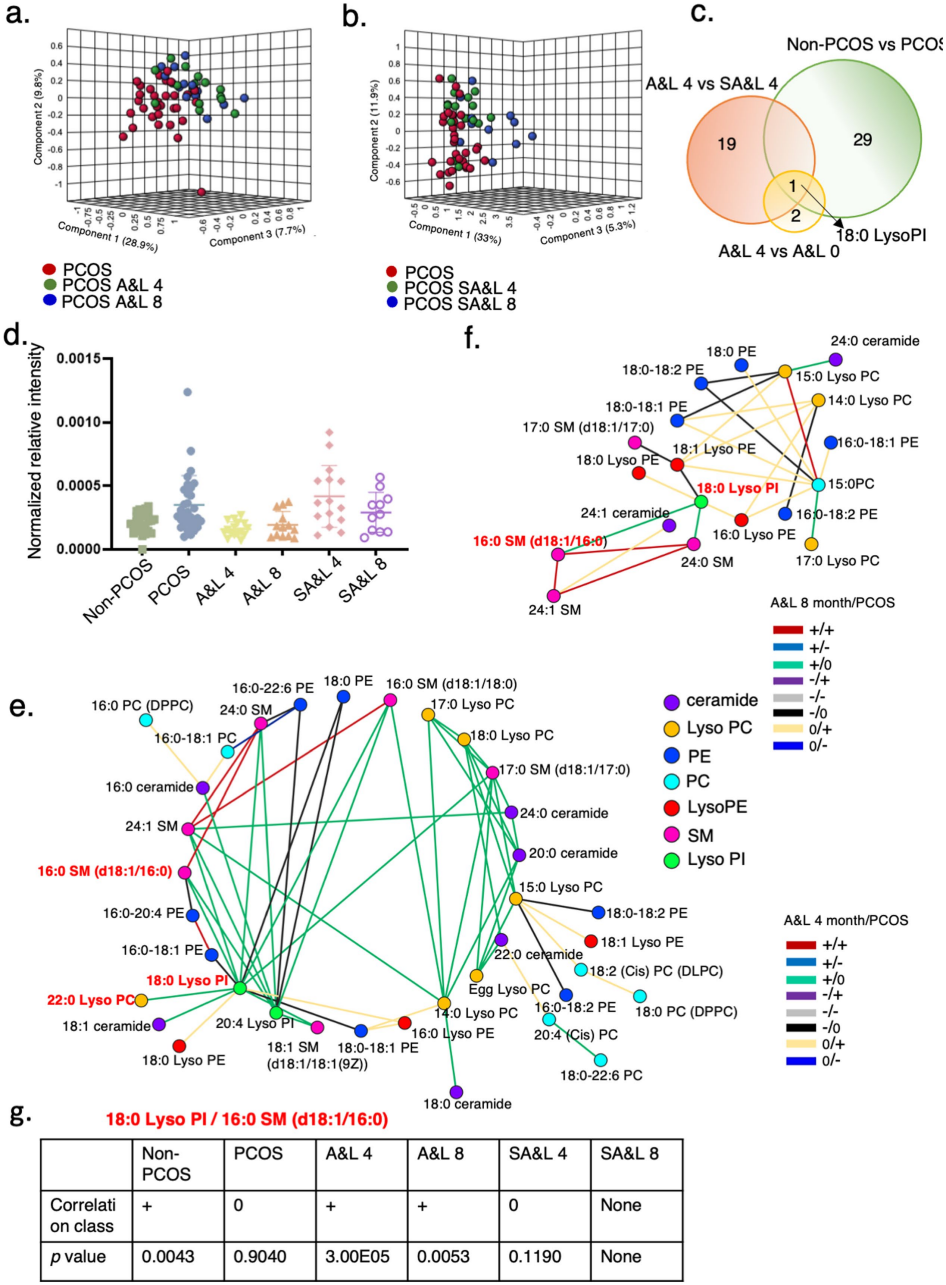
18:0 Lyso PI is associated with lifestyle and acupuncture intervention in PCOS. **(a)** PLS-DA model for distinguishing between the PCOS group before treatment, and 4 and 8 months after A&L intervention. **(b)** PLS-DA model for distinguishing between the PCOS group at baseline, and 4 and 8 months after SA&L intervention. **(c)** Venn diagrams showing overlap of significantly changed lipids in the indicated groups. **(d)** Levels of relative intensity of 18:0 Lyso PI in controls, PCOS before treatment, PCOS with 4 months A&L or SA&L intervention and these patients with another 4 months follow up. **(e,f)** Differential correlation analyses of plasma lipids in PCOS after A&L intervention versus PCOS. Multiscale embedded correlation network analysis illustrates differential correlation of lipids in PCOS after 4 months A&L intervention **(e)** and after another 4 months follow up **(f)**. Only lipid pairs with significant differential correlations (empirical *p* < 0.05) are included. **(g)** Analysis of the differential correlation variation between the lipid pair 18:0 Lyso PI and 16:0 SM (d18:1/16:0) in different groups.

The identified lipids effectively distinguished participants with PCOS before and after 4 months of the A&L intervention. However, these lipids could not distinguish participants between the 4-month A&L intervention and the 4-month follow-up point (Figure 4a), consistent with the sustained relief of PCOS symptoms observed in the 4 months follow up after the A&L intervention. Conversely, lipidomic analysis could not differentiate participants who underwent 4 months of the SA&L intervention from baseline or the 4-month follow-up point, in line with the faint improvement of PCOS symptom in the SA&L group (Figure 4b).

In the differential lipidomic analysis, 29 lipids exhibited differences between participants with PCOS and the non-PCOS control group. Additionally, 19 lipids significantly differed between the A&L intervention and SA&L intervention groups after adjusting for BMI. Among these, 18:0 Lyso PI was notable, showing significant differences of the relative intensity between participants with PCOS and the control group, as well as significant reductions after 4 months of the A&L intervention and at the 4-month follow-up point. No significant changes were observed in 18:0 Lyso PI in the SA&L intervention group before and after treatment (Figure 4d).

Lastly, 18:0 Lyso PI was positively correlated to 16:0 SM (d18:1/16:0)/22:0 Lyso PC after 4 months A&L treatment compared to no correlation in PCOS, as shown in green lines (+/0) (Figure 4e). It may be attributed to the significant reduction in the relative intensity of 18:0 Lyso PI in participants with PCOS after the A&L intervention. This positive correlationship of 18:0 Lyso PI to 16:0 SM (d18:1/16:0)/22:0 Lyso PC is similar with the control group (Figure 4g).

The positive correlation between 18:0 Lyso PI and 16:0 SM (d18:1/16:0) persisted until the end of 4 months follow up of participants with PCOS who underwent the A&L intervention, as shown by green line (+/0). The relative intensity of 18:0 Lyso PI was still significantly lower at the 4-month follow-up point after the A&L intervention, consistent with the differential correlation analysis results (Figure 4f). The correlation class and *p*-value of 18:0 Lyso PI and 16:0 SM (d18:1/16:0) in different groups are shown in Figure 4g.

## 4 Discussion

Our study provides valuable insights into the effectiveness of lifestyle & acupuncture treatment in improving symptoms of PCOS with overweight and obese. The findings highlight the potential of lifestyle & acupuncture as a cost-effective and low-risk treatment for PCOS especially in overweight and obese patients. Additionally, an in-depth lipidomic analysis identified a panel of lipid species that effectively distinguished participants with PCOS from the non-PCOS control group (AUC = 0.9747). Among these, serum 18:0 Lyso PI was observed to correlate with clinical improvement following the intervention. Additionally, coordinated changes in 18:0 Lyso PI and 16:0 SM (d18:1/16:0) were detected in response to lifestyle & acupuncture treatment. However, it is important to note that these associations do not establish causality, and further mechanistic studies are needed to clarify the role of these lipids in the treatment process.

Overweight and obesity are known to exacerbate the clinical and biochemical features of PCOS, making weight management a primary treatment recommendation (25, 26). However, current conventional medical treatments for overweight and obese with PCOS have limited effectiveness and safety (27). Appropriate diet, physical activity - lifestyle changes were crucial in the treatment of PCOS. However, their efficacy has been unsatisfactory. Acupuncture, as a complementary therapy, has shown promise in promoting weight loss through appetite and energy expenditure regulation in some studies (28). Acupuncture treatment alone has been reported to improve glucose metabolism and insulin resistance in patients with PCOS. However, it has shown little improvement in other clinical indicators in patients with PCOS (29). Our study demonstrated a significant reduction in BMI and improvements of insulin resistance, hyperandrogenism and physical activity among overweight and obese PCOS patients following a combined lifestyle intervention and acupuncture. These findings suggested that acupuncture combined with lifestyle intervention is more effective than either treatment alone. Additionally, we explored the underlying mechanisms of this combined intervention.

Multi-omics technologies—including proteomics, lipidomics, and metabolomics—have systematically characterized the molecular networks underlying PCOS. We conducted a literature review and summary across proteomics, lipidomics, and bile acid omics. Proteomic analyses identified aberrant serum levels of FSHB, IGF family proteins (including IGF-1/IGFBP-3 axis), and inflammatory markers such as S100A8/A9 (30–33). Lipidomics revealed metabolic disturbances involving triglycerides, sphingolipids (e.g., S1P in follicular fluid), and long-chain saturated fatty acids, while bile acid dysregulation was observed in follicular microenvironments (34–36). Metabolomic studies demonstrated dysregulation in fatty acids, sterols, and energy metabolic intermediates (15, 37–39). To our knowledge, the significant change of lipids 18:0 Lyso PI identified in this study has not been reported in previous studies. These multi-omics findings (Supplementary Table S2) collectively demonstrate that PCOS patients exhibited pathological features including insulin signaling pathway impairment, abnormal activity of key enzymes in androgen biosynthesis, and metabolic disturbances in follicular microenvironments.

In this research, lipidomic analysis was conducted to explore the molecular mechanisms of acupuncture intervention. Six lipid species, including sphingomyelin (SM), phosphatidylcholine (PC), lysophosphatidylcholine (LPC), phosphatidylethanolamine (PE), lysophosphatidylethanolamine (LPE), and lysophosphatidylinositol (LPI), were significantly associated with PCOS. SM, a crucial component of cell membranes, has been linked to insulin resistance and metabolic disorders (40). Lower levels of SM, PC, and LPC were observed in participants with PCOS, consistent with previous studies (34, 41), suggesting that these lipids may play a role in the disorder’s pathophysiology.

Notably, 18:0 Lyso PI was strongly associated with adiposity and exhibited significant changes in PCOS following acupuncture treatment (40). Initially elevated in participants with PCOS, the levels of 18:0 Lyso PI reduced after acupuncture treatment and remained stable at the 4-month follow-up. These changes suggest a potential link between acupuncture-mediated metabolic improvements and alterations fatty acid and lysophospholipid metabolism. However, the observed association does not imply causality, and further research is necessary to elucidate whether modulation of 18:0 Lyso PI contributes mechanistically to symptom alleviation in PCOS.

LPI is an important signaling molecule in regulating inflammation, insulin production, and insulin sensitivity. It has been reported to act through the G protein-coupled receptor GPR55 to stimulate intracellular insulin signaling pathways. To our knowledge, the co-regulation of 18:0 Lyso PI with 16:0 SM (d18:1/16:0) has been demonstrated for the first time. Thus, the effect of acupuncture on insulin resistance of PCOS may potentially be linked to the interaction of these lipid species with the insulin signaling pathway (42). A reduction in plasma and urine LPCs has been reported to indicate insulin resistance and risk of type 2 diabetes (43), but this lipid did not change after acupuncture treatment in our study. This finding suggests that acupuncture treatment did not alleviate insulin resistance by modulating Lyso PC, which may enhance understanding of PCOS disease processes and introduce new biomarkers for early diagnosis and improved patient management (44, 45).

Interestingly, the acupuncture intervention group exhibited lower levels of HbA1c, fasting insulin, and HOMA-IR after 4 months of treatment. These improvements in insulin resistance were maintained at the 4-month follow-up, indicating the long-lasting effects of acupuncture. Acupuncture has been hypothesized to act as an insulin sensitizer in previous studies by improving glucose regulation (29, 46), and our findings are consistent with these studies.

Although this study provides valuable insights, several limitations must be acknowledged. First, the absence of an independent validation cohort limits the generalizability of our findings, as the efficacy and robustness of PCOS-associated lipids may vary across diverse populations. Second, the single-center design restricts variability in patient demographics and clinical practices, potentially introducing selection bias and compromising the external validity of the results. Furthermore, the follow-up duration was insufficient: clinical data were collected only at a single time point (8 months post-treatment), which undermines the comprehensive evaluation of long-term therapeutic effects and sustainability. Future studies should prioritize multicenter collaborations, incorporate external validation cohorts, and implement longitudinal follow-ups with multiple time points (e.g., 12/24/36 months post-treatment) to strengthen the reliability of conclusions through systematic data collection.

## 5 Conclusion

In summary, our study demonstrated lifestyle and acupuncture as a feasibility and acceptability treatment in overweight/obese PCOS. The lipidomic analysis revealed characteristic changes in lipid profiles among women with PCOS, with 18:0 Lyso PI emerging as a potential factor associated with PCOS that responded to lifestyle and acupuncture therapy. This finding suggested that 18:0 Lyso PI may represent a therapeutic target in managing PCOS.

## Data Availability

The raw data supporting the conclusions of this article will be made available by the authors, without undue reservation.
