# Supplementary figures and images for: Clinical symptom improvement and lipidomic signatures in overweight/obese PCOS treated by lifestyle and acupuncture intervention
Source: Front Med (Lausanne). 2025 Oct 24;12:1642095. doi: 10.3389/fmed.2025.1642095 (PMC12592129; doi:10.3389/fmed.2025.1642095)

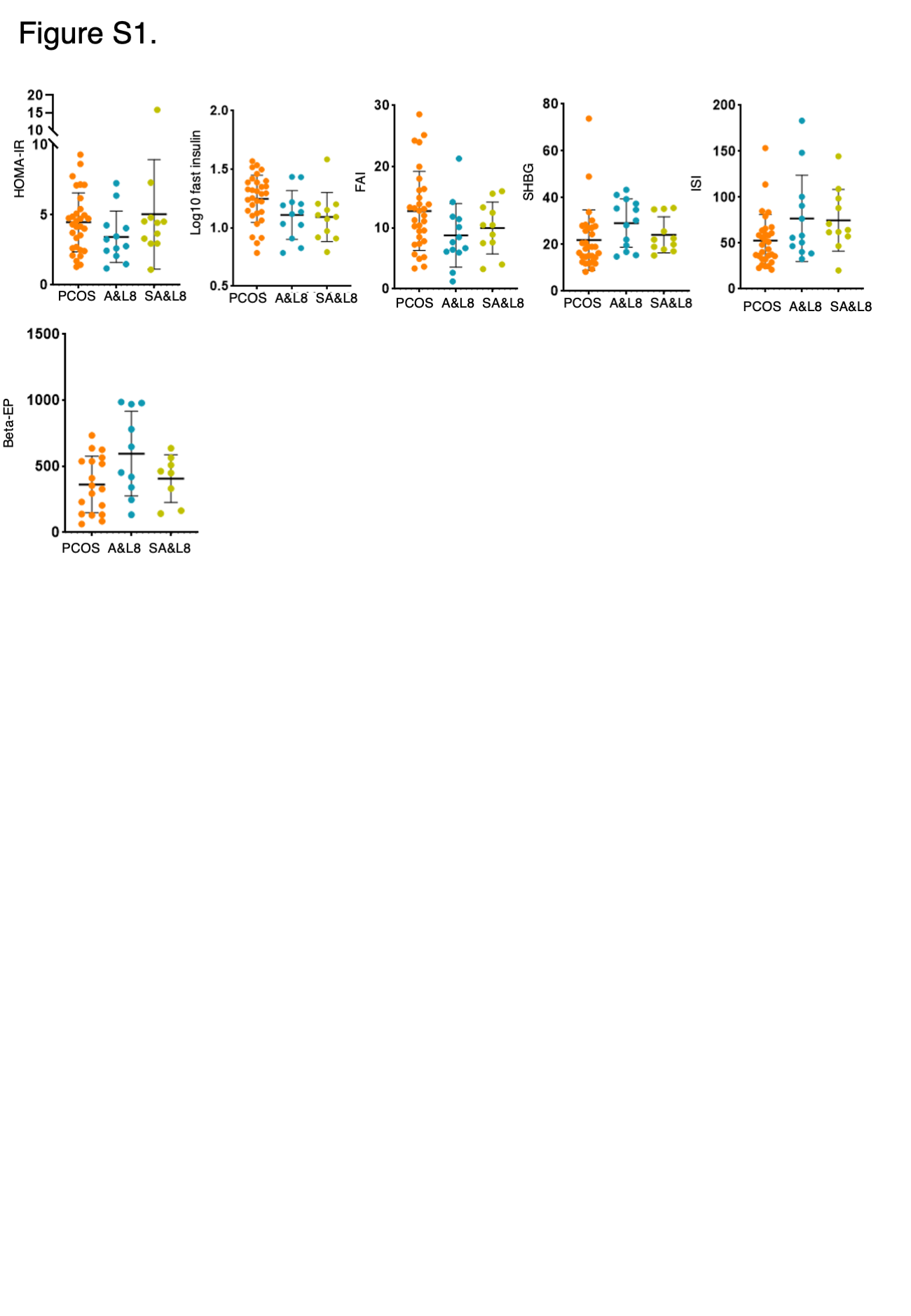

Supplement: Supplementary file 1 [file Image_1.tiff]

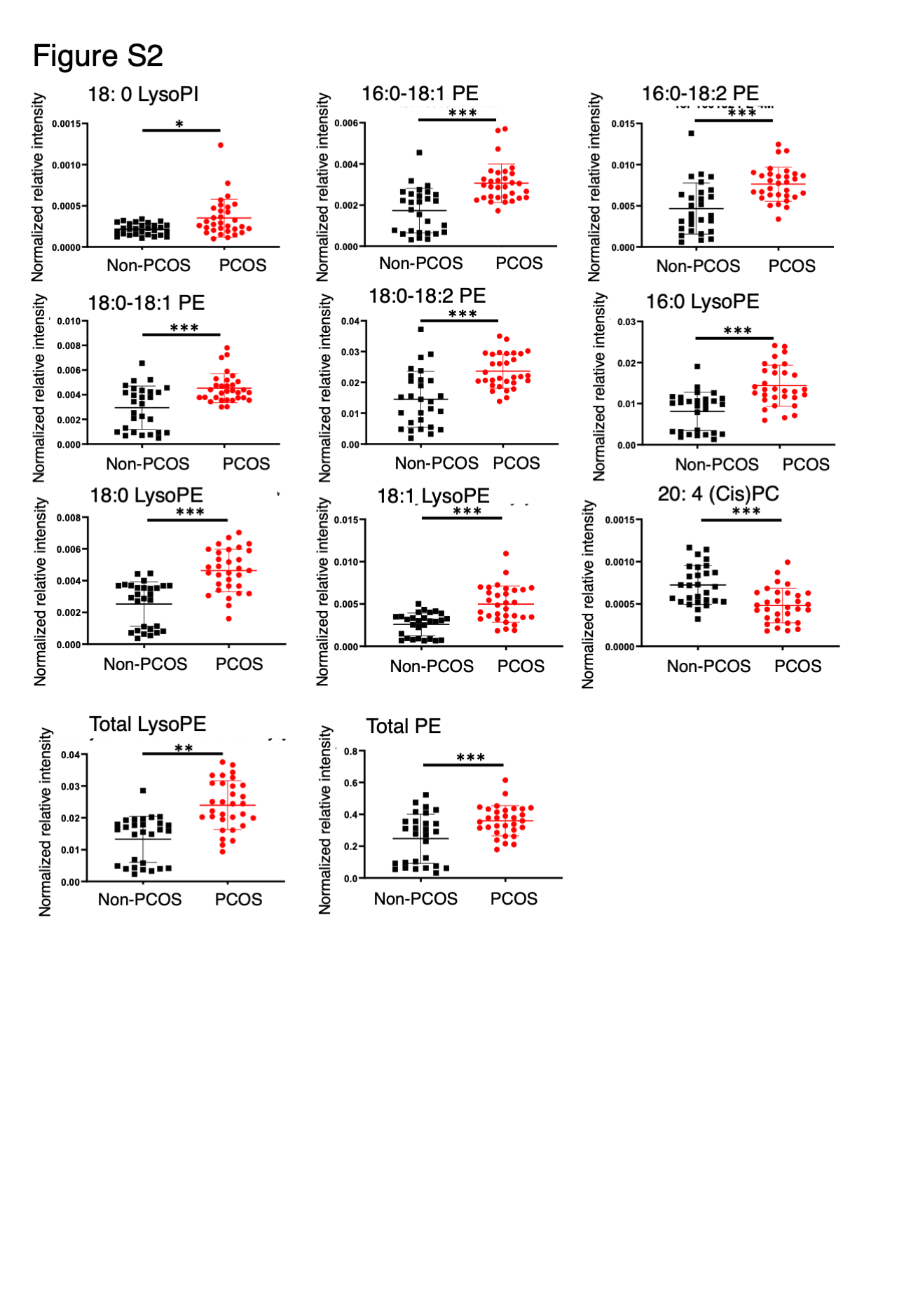

Supplement: Supplementary file 2 [file Image_2.tiff]
