# Supplementary material for: Clinical symptom improvement and lipidomic signatures in overweight/obese PCOS treated by lifestyle and acupuncture intervention
Source: Front Med (Lausanne). 2025 Oct 24;12:1642095. doi: 10.3389/fmed.2025.1642095 (PMC12592129; doi:10.3389/fmed.2025.1642095)
Supplement: Supplementary file 4 [file Table_2.docx]

**Table S2.** Multi-omics technologies (including metabolomics, lipidomics, and proteomics) have elucidated the complex molecular networks underlying polycystic ovary syndrome (PCOS).

| **Analyses** | **Disease** | **Detected molcules** | **Samples** |
| --- | --- | --- | --- |
| Proteomics | PCOS | FSHB, SIGLEC12, FUT10, NUCB2, NCR3LG1, C1QTNF9, EGLN1, MAPK9, SBSN, DB, MRI, UROD(30) | Serum |
|  | PCOS | Apolipoprotein C3(ApoC-III),  S100A8/A9, Complement C3, CD14, Afamin, Serotransferrin, A1BG, IGF-1, IGF-2, IGFBP-2, IGFBP-3, IGFBP-4(31, 32) | Serum |
|  | PCOS and T2DM | PKM1/M2, ApoA-I, Albumin, Peroxiredoxin 2, Annexin A2, A1BG, Flotillin-1, Haptoglobin(33) | Serum |
| Lipidomics | PCOS | glycerides, glycerophospholipids, sphingomyelin(34) | Follicular Fluid |
|  | PCOS and obesity | lysophospholipid, PUF and long-chain saturated fatty acids(35) | Serum |
|  | PCOS | Triglycerides, sphingomyelins, lysophosphatidylcholines and phosphatidylethanolamines(36) | Serum |
| Metabonomic | PCOS Women with Insulin Resistance | stearic acid, palmitic acid, pentadecanoic acid, stigmasterol, citric acid, isocitric acid, thymine, and pyruvic acid  Lithocholic acid and sinapinic acid(37) | Follicular Fluid |
|  | PCOS | Glycocholic acid (GC3A), taurocholic acid (TCA), glycochenodeoxycholic acid (GCDCA), and chenodeoxycholic acid-3-β-d-glucuronide (CDCA-3Gln)(38) | Follicular Fluid |
|  | PCOS | phosphatidylglycerolphosphate (PGP), a triglyceride (TG), lysoPE, PC, sphingolipids, glycerophospholipids and fluctuated fatty acyls(39) | Follicular Fluid |
|  | PCOS | S1P, Cer, SM, SM species with long saturated acyl chains, LacCer(15) | Serum |
