# Supplementary material for: Clinical symptom improvement and lipidomic signatures in overweight/obese PCOS treated by lifestyle and acupuncture intervention
Source: Front Med (Lausanne). 2025 Oct 24;12:1642095. doi: 10.3389/fmed.2025.1642095 (PMC12592129; doi:10.3389/fmed.2025.1642095)
Supplement: Supplementary file 5 [file Supplementary_file_1.docx]

**Figure S1.** **Changes of** **the clinical indices in PCOS patients after 8 months acupuncture & lifestyle intervention and sham acupuncture & lifestyle intervention.**

The clinical indices in PCOS patients improved after 8 months acupuncture & lifestyle intervention, but there was no significant difference.

**Figure S2.** **Nine differential lipids with fold change greater than twice.** The levels of relative intensity of nine significantly changed lipids between patients with PCOS before treatment and healthy controls with the criteria of adjust *p* value < 0.05 and a fold change above 2. Values are presented as the mean with the standard deviation (SD).
